# Building a 3D Virtual Liver: Methods for Simulating Blood Flow and Hepatic Clearance on 3D Structures

**DOI:** 10.1371/journal.pone.0162215

**Published:** 2016-09-20

**Authors:** Diana White, Dennis Coombe, Vahid Rezania, Jack Tuszynski

**Affiliations:** 1 Department of Mathematics, Clarkson University, Potsdam, New York, United States of America; 2 Computer Modelling Group Ltd, Calgary, Alberta, Canada; 3 Department of Physical Sciences, MacEwan University, Edmonton, Alberta, Canada; 4 Department of Physics and Department of Oncology, University of Alberta, Edmonton, Alberta, Canada; University of Colorado, UNITED STATES

## Abstract

In this paper, we develop a spatio-temporal modeling approach to describe blood and drug flow, as well as drug uptake and elimination, on an approximation of the liver. Extending on previously developed computational approaches, we generate an approximation of a liver, which consists of a portal and hepatic vein vasculature structure, embedded in the surrounding liver tissue. The vasculature is generated via constrained constructive optimization, and then converted to a spatial grid of a selected grid size. Estimates for surrounding upscaled lobule tissue properties are then presented appropriate to the same grid size. Simulation of fluid flow and drug metabolism (hepatic clearance) are completed using discretized forms of the relevant convective-diffusive-reactive partial differential equations for these processes. This results in a single stage, uniformly consistent method to simulate equations for blood and drug flow, as well as drug metabolism, on a 3D structure representative of a liver.

## Introduction

The liver is the central metabolic organ in human physiology, being responsible for the biotransformation of endogenous and toxic substances in the body.

As such, one of its main roles is to prevent the accumulation of certain chemical compounds in the blood by converting them into a form suitable for elimination. Various compounds are delivered into the liver by blood flow through the portal vein and hepatic artery, and are processed by the hepatic cells in the liver’s tissue. Metabolism by enzyme-catalyzed biotransformation produces chemical alterations in the original compounds so that they can be eliminated. Because of its role in drug elimination, when the liver is not healthy, or if the administered drugs are too toxic, individuals may become very sick or die. In fact, liver toxicity is a primary reason for the failure of a significant proportion of many promising drugs that consequently never reach the market, and so realistic models for hepatic clearance are important. As such, computational models, such as the one we will present in this paper, can give experimentalists a better idea about which chemical compounds might be better to test in terms of their toxicity. Such information could save time and money, as certain classes of chemicals could be eliminated from testing before lengthy and expensive clinical trials begin.

In this paper, we describe a physiologically based modeling approach that can be used to explicitly describe blood and drug flow through a liver at the organ scale, as well as describing the uptake of drugs at the tissue level, and their eventual elimination. Such studies have previously been completed at the lobule scale [1–5], but there is a lack of realistic models that describe the time-resolved distribution of compounds within the whole organ (in both the large-scale vasculature, as well as the tissue). Many of the models that measure flow at the organ scale do not take into consideration the complex spatial organization of vessels in the liver. For example, pharmacokinetic (PK) models [6] are compartmental-type models, used to measure flow on a predefined number of individual compartments, where each compartment has a unique set of physiological parameters to describe it. Although these models can provide insight into averaged processes happening at large scales, they cannot capture the full complexity of the liver, such as tracking mass through fine vasculature structures and describing metabolism at the scale of the lobule.

Other models, based on electrical analogues, have been used to describe hemodynamics through the liver [7] [8]. Such models are used to study internal pressures and blood flow rates in the liver during hypothermic machine perfusion (HMP). HMP is a technique used to perfuse organs that are being prepared for transplantation. Such electrical models not only provide information about pressures and flow rates, they also include information about vessel lengths and radii. Although these models are useful at describing HMP, they are not suitable to describe hepatic clearance over the liver, as they do not include information regarding the spatial organization of the vessel segments. In particular, there is no description of the spatial distribution of flow. Also metabolism is not a component of this modeling approach.

More recently, a spatio-temporal model has been developed, using *in vivo* micro-CT imaging, to generate geometrically accurate vasculature structures for mice [9]. Here, the authors complete simulations on two vasculature structures (which they call the supplying and draining structures), to describe blood and drug flow, and to simulate metabolism at the cellular level. In this model, fluid and drug flow through each vasculature structure is described as a completely convective process. Once the flow reaches the end of the input supplying vasculature (the portal vein structure), averaged species concentration values are used as the input flow values for their “homogenized hepatic space” (HHS), the tissue component of the model. From liver images, their full liver is discretized into a collection of tissue HHS’s with possibly variable reactive metabolic parameters. This allows for a non-homogeneous collection of HHS and thus some spatial variability of drug metabolic responses. Each HHS is composed of several subspaces in analogy to the liver compartments used in other PK models. The sub-compartment that implicitly describes the sinusoids is treated as a porous medium, and so flow is calculated according to Darcy’s Law. After leaving the HHS, locally averaged concentrations of components of interest flow back through the output draining vasculature (the hepatic vein) and the flow rates and concentrations of each component (e.g., blood or drugs) are predicted and compared with measurements. In essence however, their method consists of two distinct modules for vasculature and tissue, where each vasculature is coupled in series to the tissue space through an averaged transfer mechanism.

Similar to the above approach, we simulate fluid flow over two vasculature structures (a portal vein and hepatic vein structure). The main advantage of our approach, compared to that described in [9], is that we implicitly and consistently, within a single stage model, solve equations for metabolism and flow at each grid cell that surrounds the vasculature. Computationally, the collection of HHS described in [9] is much fewer than the number of tissue grid cells used in our model (see S2 Appendix) and we account for flow and reaction in each tissue grid cell individually. Unlike the model described in [9], we do not generate a fully 3D vasculature. In particular, as a first step, we generate 3D vasculature structures with branching angles in 2D. We do so to avoid vessel overlap between the two vasculature structures, which can produce an unphysiological shunt (a potential shortcoming of the model described in [9]). We realize that stacking of the two vasculatures leads to a somewhat artificial vasculature description. We note the assumption of [9] that the two vasculatures are in series with tissue is essentially equivalent to our stacked vasculature system coupled through a tissue phase however. Our objective here is to describe our methods approach, while more realistic 3D vasculature structures will be used in future studies. Presently, we are able to generate fully 3D portal and hepatic vein vasculatures separately. However, we are still in the process of generating both vasculatures simultaneously without there being any vasculature overlap. An example of a 3D portal vasculature structure, generated on a liver-shaped domain, is illustrated in the S1 Appendix and compared with a portal vasculature from the literature.

The overall goal of this project is to come up with a novel methodology for describing blood and drug flow through the complex architecture of the liver vasculature system, as well as a methodology for describing drug uptake and elimination at the tissue level. Thus, for simplicity in understanding the approach, as well as removing the difficulty of generating two non-overlapping vasculature simultaneously (a potential limitation of other models [9]), we start by modeling the branching angles for vasculature in 2D, and illustrate the model with numerical simulations.

The Methods and Models section consists of four main parts. First, we describe a method to generate 2D images representing the portal vein and hepatic vein vascular structure, using a computational algorithm based on the existing method of computational constructive optimization [10] [11]. For the generation of the 2D portal and hepatic vasculature, we use input data representative of a dog liver, since such information has been well documented in the literature [7] [12] [13]. Next, we describe a novel computational method for generating a 3D structure, from the 2D images, representing an approximation to a liver. We then describe the method used to model blood and drug flow, as well as drug metabolism, on the generated 3D structure, based on a finite difference reactive fluid flow model STARS [14]. In the final section of Methods and Models, we describe a method for calculating fractal properties for our generated vasculature. Such properties have also been described for real liver vasculature. As our model is not yet 3D, fractal-type analysis such as this is a useful method to validate our results with other 2D studies. In the Results Section, we highlight how our model works by illustrating simulation results for hepatic clearance. Finally, in Discussion and Conclusions, we discuss the advantages and limitations of our modeling approach, and how we aim to improve our treatment in future studies.

## Methods and Models

### Generation of 2D vascular structure using the method of CCO

The large-scale architecture of blood flow in the liver comprises of three main vasculature structures: two supply vasculature, the portal vein and the hepatic artery, and one draining vasculature, the hepatic vein. Approximately ¾ of all blood coming into the liver comes from the portal vein, which is comprised of blood coming from the spleen, gastrointestinal tract, and its associated organs [15]. The final ¼ of incoming blood comes through the hepatic artery, which carries oxygenated blood from the heart [15]. The vessels from these supply trees bifurcate down into the tissue bed (lobules), where nutrients and metabolites contained in the blood are metabolized. Once blood leaves the lobules, it exits through the third vasculature structure, the hepatic vein [15]. At the lobule scale, several imaging techniques (scanning electron microscopy, confocal laser scanning, etc D’Alessandro et al, [16]; Hoehme et al [17]) can be used to quantify sinusoid structures.

In past work, vascular structures at the full organ scale have been generated using a number of different approaches. One approach is the construction of vasculature structures from real images [18], and another is to construct vasculature structures using fractal-type models [19]. Vasculature structures constructed from real images can lack fine details because imaging techniques (such as CT or MRI scans) can only describe vasculature down to a certain size. Also, these methods are essentially 2D, where a 3D construction is generated by stacking many 2D images together. For such methods, many of the very small vessels leading to the tissue cannot be described. Fractal models, on the other hand, can be used to describe very small vessels, and can generate their appropriate radii and lengths. However, in many of these models, a description of the spatial arrangement of such segments is lacking.

In an effort to combine a description of realistic vessel size with a description of the proper spatial locations of all vessel segments at the organ scale, the method of constrained constructive optimization (CCO) is used [11] [10] [20] [21] [22]. Such a method was initially used to describe the vascular structure of a 2D cross-section of the heart (by approximating the heart’s cross-section as a circular domain), and allows for the generation of very detailed vascular structures. Resulting vasculatures generated using this method have been tested against morphometric data and results have shown similarities with real arterial trees [11]. More recently, this method has been extended to three dimensions [23], and can be used to generate vasculature on numerous types of organs [24], as well as many other complex shaped domains [25].

The concept of CCO is to build a realistic vascular structure, from first principals, which supplies blood evenly throughout some prescribed domain in an optimal way (i.e., the primary assumption is that blood is perfused homogeneously). In particular, all terminal segments of a vasculature tree should be distributed homogeneously as possible throughout a given domain, so as to deliver an equal flow of blood *Q*_term_, as well as to exert a unique terminal pressure *P*_term_, through each terminal segment. Here, we briefly outline the generation of a single vasculature tree and leave it to the reader to look through earlier works for a more detailed description [11].

#### Initialization of the tree

First, a root point (the entry location for blood into an organ) is chosen on the surface of the domain. Here, as a first approximation, we choose our domain to be a simple elliptical structure.

Next, a second (random) point is selected within the interior of the domain, defining the first terminal node of the tree structure. The connection of the root point to this point defines the root segment. The radius *r* of the root segment is calculated using Eq 1, so as to satisfy the total resistance of the root segment *R*_tot_. This total resistance is kept constant throughout the entire building process. In particular, *r* is calculated by assuming that the root segment (and each segment that follows) is represented by a cylinder and perfused according to Poiseuille’s Law so that total resistance *R*_tot_ is given by

$$R_{tot}=\frac{8\eta l_{root}}{\pi {r_{root}}^{4}},$$ (1)

where *η* is the viscosity of blood and *l* is the segment length.

After the root segment is defined, a third point is selected at random within the domain, representing the second terminal node of the vasculature tree. A distance criterion must be satisfied to make sure that both terminal nodes are sufficiently far from one another. If the criterion is not satisfied, a new random point is selected. This process is repeated N times. If the distance criterion is not satisfied after N times, it is weakened slightly and the selection process repeats itself. Again, if no suitable point is found after N draws, the criterion is weakened again. This process repeats until a suitable second terminal node is found.

Once a second terminal node is found, it is connected to the midpoint of the first (and only) segment. This connection is referred to as a bifurcation point, and the radii of the three newly formed segments are scaled according to the bifurcation equation

$${r_{p}}^{\gamma }={r_{l}}^{\gamma }+{r_{r}}^{\gamma },$$ (2)

which has been derived from morphometric analysis of real arterial trees [26]. Here, *r*_*p*_ is the radius of the parent (root) segment, *r*_*l*_ is the radius of the left daughter segment, and *r*_*r*_ is the radius of the right daughter segment. The parameter *γ* can be any parameter greater than zero. However, for real arterial trees, direct measurement at bifurcations gives a value of *γ* = 3.0 [27]. Such a value has been used in the construction of other vasculature trees, and corresponds to a necessary condition for equal stress between blood and the vessel walls in both input and output branches [10]. Alternatively, *γ* = 2.55 was obtained indirectly by counting the number of end branches. This value allows for minimum reflection of pulse waves [28].

To determine the value for each segment radius, we use the fact that the pressure drop *ΔP* along each segment satisfies an Ohm’s-type relation

$$\Delta P_{i}=R_{i}Q_{i},$$ (3)

where *R*_*i*_ is the resistance through a segment *i* and *Q*_*i*_ is the flow through that segment. We also use the fact that the flow through each segment satisfies Poiseuille’s Law

$$R_{i}=\frac{8\eta l_{i}}{\pi {r_{i}}^{4}},$$ (4)

where *l*_*i*_ is the length of segment *i* and *r*_*i*_ is the radius. Using the fact that the pressure drop and the flow rate along each terminal segment are equal, we can use Eq 4 and write

$$\frac{l_{l}}{{r_{l}}^{4}}=\frac{l_{r}}{{r_{r}}^{4}}.$$ (5)

From Eqs 2 and 5, and writing the total resistance of the tree as the sum

$$R_{tot}=R_{p}+{\left(\frac{1}{R_{r}}+\frac{1}{R_{l}}\right)}^{-1},$$ (6)

we can calculate each of the three unknown radii.

After the three radii are calculated, the connection point, which we refer to as a bifurcation point, is moved along an optimal path so as to minimize the total volume of the structure. In particular, for a tree consisting of one bifurcation, the total volume of the tree is given by

$$V=\pi \sum_{i=1}^{3}r_{i}^{2}l_{i},$$ (7)

and the optimal path is directed towards the negative gradient of the total volume (starting at the bifurcation point). We use the method of steepest descent for the minimization routine [29]. After each step along the optimization path, the radii are rescaled according to the Eqs 2, 5 and 6, and a new total volume is calculated. Optimization stops only when a minimum total volume is reached (i.e., the difference between successive volume calculations becomes very small, reaching a defined threshold. For the simulations presented here, the threshold is set to 10^-7^cm). The minimized structure, containing three segments, is the starting structure for the vascular tree.

#### Adding to the initial tree

After initializing the tree structure, new segments are added until the appropriate number of pre-defined bifurcations is reached. For each addition to the tree, a new random point is selected. This point must be sufficiently far from all other terminal nodes (as described above). Once such a point is found, a connection is made between it and the midpoint of each segment in turn (we do not consider segment connections that cause segment crossings). For each connection to a segment, the structure is optimized using the same method as described above. As before, along each step in the minimization process, the radii of all vessels in the tree are rescaled at each step in the minimization process, according to Eqs 2 and 5, and a generalization of Eq 6. For example, if we connect the third terminal node to the left segment of the initialized tree (the tree with three segments and two terminal nodes), *R*_*l*_ in Eq 6 becomes $R_{l}=R_{p2}+{\left(\frac{1}{R_{r2}}+\frac{1}{R_{l2}}\right)}^{-1}.$ Here, *R*_*p2*_ is the new root segment, and *R*_*r2*_ and *R*_*l2*_ are its corresponding left and right daughters. A good description for how this equation generalizes in described in [23].

Once a minimized structure is found for a particular segment connection, the total volume is recorded. Then, the next segment connection is made and the optimization process repeats itself. Once all segment connections have been tested, the overall minimum volume structure is chosen. A new terminal node is selected and the process repeats itself. The algorithm is outlined in Box 1 below.

**Box 1. Outline of algorithm for method of CCO**

Summary of Algorithm

1. Choose the input root location on the boundary of the domain.
2. Randomly select the first terminal node and calculate the radius. The radius is defined by the total input flow and input and terminal pressures.
3. Select a new terminal node (make sure it is far away from all other terminal nodes) and connect it to midpoint of each existing segment in turn.
4. Minimize each segment connection accordingly and record total minimized volume.
5. Once all segment connections have been minimized and the minimum volumes recorded, choose the lowest volume structure.
6. Continue the process until the total number of desired bifurcations is attained.

**Fig 1:**
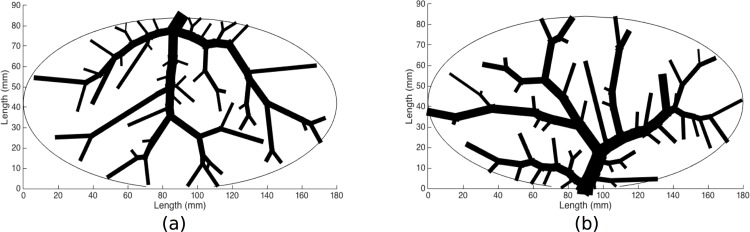
Examples of 2D vasculature generated using method of CCO on an ellipse (ellipse removed). (a) Portal vein and (b) hepatic vein, for 50 bifurcations.

Fig 1 describes a 2D representation of a portal vein and hepatic vein vasculature structure generated by CCO on an ellipse using input data for medium sized dogs (See Table 1). The reason we choose to use data for dogs is that such information is well documented in the literature [12] [28]. For simplicity, we neglect the construction of the hepatic artery since the majority of the blood coming into the liver comes from the portal vein. Also, both the portal vein and hepatic artery vasculature essentially run in parallel, and so combining them as a first approximation seems reasonable. We should also mention that, for realistic flow results, this method should be extended so that branching angles are defined in 3D. As stated previously, it is difficult to produce two non-overlapping vasculature simultaneously in 3D, and this is the focus of our future work. Also, a comparison of the generated vasculature structures should be compared to experimental data. In particular, the branching angles and vessel radii should be compared to data from CT scans, as was done in [30], or data from corrosion casts, as was done in [30] and [31].

**Table 1:** Input parameters used in the CCO generation of the vasculature structures shown in Fig 1.

Parameter	Portal Vein	Hepatic Vein	Meaning and Source
*ΔP*	15Pa (0.11 mmHg)	10Pa (0.09 mmHg)	Pressure (this paper)
*Q*_tot_	240ml/min	390ml/min	Total blood flow [7]
*r*_*0*_	0.55 cm	0.45 cm	Root radius (calculated using Eqs 3 and 4)
*BifNum*	50	50	Number of bifurcations
*γ*	3	3	Bifurcation parameter

The input data used in the creation of each vasculature are summarized in Table 1.

The cross-sectional length of the ellipse is chosen to be 18cm and the cross-sectional width is chosen to be 8.4cm. We choose these values so as to obtain a total volume for the generated 3D structure (described in the next section) that is approximate to the volume of a dog liver. For simplicity, we chose the structure of an ellipse to approximate our domain boundary. For a real liver, the largest cross-sectional distance along the horizontal axis (craniocaudal axis) is always larger than the largest cross-sectional distance along the width (midclavicular line) of a liver. Here, we imagine that the cross-sections through the center of the ellipse, in the horizontal and vertical direction, are representations of the cross-sectional length and width of a real liver (see Fig 1 for a description of these distances).

The flow rates used in the creation of the vasculatures illustrated in Fig 1 are similar to flow rates found in dog livers [7]. However, the pressure drops *ΔP* are very small (see Table 1) and not realistic, and were chosen in order to obtain the desired root radius for each vasculature structure [7]. The small value for the pressure drops can be partially explained by the choice of the bifurcation parameter γ, as well as the number of bifurcations used in the generation of the vasculature (see the discussion section for more details). We have generated vascular trees in 2D using lower values of γ and more vessel segments, and have found that more realistic values for the pressure drop can be used to obtain similar desired root radii (results not shown). In general, for dogs, the pressure drops are approximately 5mmHg and 3.5mmHg for the portal vein and hepatic vein vasculature, respectively [7].

### Transforming 2D liver representation to a lattice-based quasi-3D liver approximation

**Fig 2:**
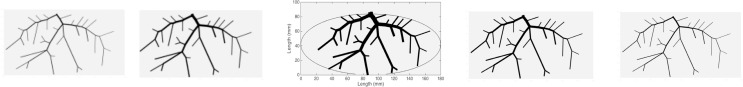
An example of a series of 2D images used in the creation of a quasi-3D vasculature structure. The central image represents a cross-section of the vasculature, while the images directly to the left and right illustrate the same image with a constant reduction in vessel diameter for all vessels. Similarly, the images to the extreme left and right show a further reduction in all vessel diameters.

To generate a 3D representation of each 2D vasculature structure described in the last section, we make use of image capturing techniques in Matlab [32]. To do this, we take a series of images of the cross-sectional vasculature structure, where each image differs by having a reduced value in the width of the vessel segments. In particular, for each image, we reduce the width of all segments by a constant value, which we denote as *iΔz*. The first image will have a reduction in width of *Δz*, the second image will have a reduction in width of 2*Δz*, and so on. An example of this image capturing technique is illustrated in Fig 2. We continue the image capturing process to *i* = *N*, at which point there is no vasculature remaining to describe (i.e., the largest vasculature segment is fully defined). We then stack these images to the top and to the bottom of the cross-sectional image (like a sandwich), so that there are 2*N*+1 layers. The value 2*N*+1 will represent the number of grid cells we will have in the z direction for our quasi-3D liver structure, where *Δz* is the grid cell length in that direction. In the rest of the paper, we will use the term “3D” to indicate “quasi-3D”, recognizing that our construction is to demonstrate our method and not to represent a true 3D liver vasculature tree.

**Fig 3:**
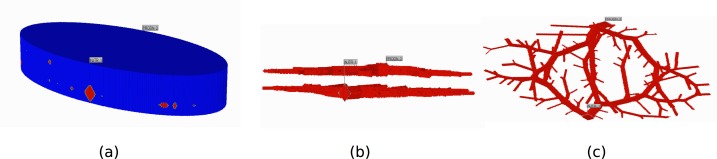
3D structure for the layered elliptical liver model. Red represents the vasculature and blue represents the tissue space. (a) The entire liver (mostly covered with tissue cells). ‘Inject_1’ represents the input location of the portal vein root and ‘Prodn_2’ represents the output location of the hepatic vein root. (b) The two vasculatures shown as a cross section through the liver. Here the tissue grid cells are removed to visualize the vasculature alone. (c) Top view of figure (b).

Next, we define the lengths of the grid cells in the x and y direction. For each 2D image created, we define the size of each image pixel in the x and y direction as *Δx* and *Δy*, respectively. Thus, at the end of the image capturing and stacking process we obtain a 3D gridded structure with grid cell dimensions *Δx*, *Δy*, and *Δz*. The process is analogous to the method of CT scanning, where a series of equally spaced cross-sectional images of a specimen are captured along a predefined vertical axis to create a 3D representation. An illustrative example of this process, with arbitrary dimension size *Δz* is shown in Fig 3. In general, depending on the value of *Δz*, there can be many more layers. For our simulations, *Δz* = 0.04 cm, *Δx* = 0.04 cm, and *Δy* = 0.04 cm, and so all grid cells in our structure are represented by cubes.

As stated previously, we only generate vasculature structures for the portal and hepatic vein. To do this, we repeat the image capturing technique described above twice, once for each vasculature (using the same values for *Δx*, *Δy*, and *Δz*). The first set of layers produced (p layers) will represent the layers of the portal vein structure, and the second set of layers produced (the next h layers, which are stacked directly on top of these p layers), will represent the layers of the hepatic vein structure. There will be a total of p + h layers in the z direction. The full 3D structure for the layered elliptical domain is shown in Fig 3, which has a total of 54 layers in the z-direction. Red represents vasculature grid cells and blue represents the tissue grid cells.

The overall volume of the structure shown in Fig 3 is 256.4cm^3^, an appropriate size for a medium sized dog liver [13] [7]. The total porous volume, which describes the volume of the vasculature plus the fluid content of the tissue, is 84.7cm^3^. Also, as summarized in Table 1, the root radii for the portal and hepatic vein are 0.45cm and 0.55cm, respectively. These values are those reported for a dog liver by Plaats et al. [7].

### Modeling fluid flow and drug metabolism

#### Equations for fluid flow

To simulate fluid flow on the 3D liver structure described in the previous Section, we utilize the fluid flow software STARS [14]. This software is designed to model oil and gas flow through porous media, and uses a finite-difference modeling approach to solve the equations of fluid flow on various gridded structures. STARS is well suited to study blood flow through the liver, because the liver is a porous organ. Recently, STARS has been successfully used to describe blood flow and drug metabolism at the lobule scale [2], and to describe reactive flow processes on both mechanically loaded cortical bone [33] [34] and intervertebral disks [35].

In our model, we define both the vasculature and tissue as porous media, and so convective molar flux is modelled according to Darcy’s law [36]

$$J_{ik}^{c}=\rho x_{i}v_{k}=\rho x_{i}\frac{K_{k}}{\mu }\nabla_{k}p.$$ (8)

Here, $J_{ik}^{c}$ is the *i*^th^ component of fluid flow in the *k*-direction, where *c* stands for the fact that this component describes convective flow. Also, *ρ*, *μ*, *v*_*k*_, *K*_*k*_, and ∇_*k*_*p* are the fluid molar density, viscosity, Darcy velocity, permeability, and pressure gradient in the direction *k*, respectively. Parameter values for *ρ* and *μ* are outlined in Table 2. According to Darcy’s law and the convection of flow in porous media, we define the permeability *K* as the transmissibility of a grid cell face to the flow of fluid, and so this parameter has units of cm^2^. To run fluid flow simulations using this approach, we must define values for permeability and porosity for each grid cell in our layered structure. Vasculature and tissue grid cells will have different porosity and permeability value, and these values are summarized in Table 2. Note that reaction parameters are based on the work of Vaclavikova et al. [37]. In the following, we outline how to calculate these values.

**Table 2:** Base case flow and metabolism parameters for the layered liver model.

Parameter	Value (SI units)	Value (STARS unit)	Meaning
*μ*	3.5 × 10^−3^ Pa•s	3.5 cpoise	Blood viscosity
*ρ*	55.4 × 10^−6^ m^3^	55.4 mmoles/cm^3^	Blood molar density
*ϕ*_*vasc*_	0.9	0.9	Vasculature porosity
*ϕ*_*tiss*_	0.2980	0.2980	Tissue porosity
*K*_*vasc*_	5.0×10^−9^ m^2^	5.0×10^6^ mD	Vasculature permeability
Calculated *K*_*tiss*_	1.44×10^−9^ m^2^	1.44×10^5^mD	Tissue permeability
Simulated *K*_*tiss*_	2.5×10^−9^ m^2^	2.5×10^6^ mD	Increased tissue permeability
*Δx*, *Δy*, *Δz*	0.0004 m	0.04 cm	Grid spacing (in x, y, and z)
*D*_*vasc*_	4.2×10^−10^ m^2^/sec	2.5 ×10^−4^ cm^2^/min	Effective diffusion in vasculature
*D*_*tiss*_	8.4×10^−11^ m^2^/sec	5.0 ×10^−5^ cm^2^/min	Effective diffusion in tissue
*v*_*max*_	8.88×10^−4^ μM/s	9.6 ×10^−10^ molfrac/min	Maximum reaction rate
*K*_*m*_	10 μM	1.8×10^−7^ molfrac	Half saturation constant
*V*_*max*_*/K*_*m*_	8.88×10^−5^ s^-1^	5.33×10^−3^ min^-1^	Linear rate of reaction

*Vasculature Porosity* is a measure of the total volume of the vasculature in a grid cell to the total volume of the grid cell. That is, porosity ϕ is given by

$$\phi =\frac{\text{Total volume of vasculature in grid cell}}{\text{Total volume of grid cell}}.$$ (9)

Here, each grid cell is comprised completely of either vasculature or tissue, and so the porosity, as we describe above, should be given a value of 1 where there is only vasculature. However, in reality, we know that the edges of each vessel should be rounded, and so (square) grid cells near the boundary of a vessel should have a slightly smaller value for porosity than the grid cells on the interior of the vessel. To approximate this *edge effect*, the porosity of all vasculature grid cells is taken to be *ϕ*_vas_ = 0.95.

*Tissue Porosity* in grid cells is a little more complicated to define. Here, square grid cells represent lobules at our scale of interest. Lobules, the building blocks of the liver, are (roughly) hexagonal prisms, where blood flows in through portal venules [38]. Blood then travels through the lobule towards its center through very tiny vessels called sinusoids, where it is eventually dumped back out into a single central hepatic venule. Again, we emphasize that we are not explicitly considering the microstructure (and hence detailed blood flow) for each lobule in this modeling framework. Instead, we consider single grid cells to represent lobules at our scale of interest, which in turn are comprised of blood carrying sinusoids. To define the porosity of such “lobule” cells, we must consider a weighted average of both sinusoid and tissue properties. We consider the porosity of the largest single tube (st) that can be inscribed in one grid cell with dimensions of equal length, which we will denote as *d*. From Eq 9, the porosity of such a grid cell is equal to

$$\phi_{st}=\frac{\pi {\left(d/2\right)}^{2}d}{d^{3}}=0.7854.$$ (10)

The remaining porosity of the grid cell is 1–0.7854 = 0.2382 (which corresponds to a composition of sinusoidal and non-sinusoidal tissue space). Assuming that the sinusoids take up roughly 1/9 of this space, while the tissue takes up 8/9 of this space, the porosity of the tissue grid cells is written as *ϕ*_tiss_ *=* 1/9 × 0.7854 + 8/9 × 0.2382 = 0.2980. A similar definition for porosity is described by Rezania *et al*. [2] and comes from the fact that, in rat livers, sinusoid lumina represents 11% of lobule volume while the rest is represented as tissue [39].

*Vasculature Permeability K* is calculated from the radius *r* of a vessel segment (assuming that vessels are cylindrical in shape) by the equation

$$K=r^{2}/8.$$ (11)

As stated above, we choose our grid size to be 0.04cm (in all 3 directions), and so we view each grid cell to represent a tube of radius 0.02cm. From Eq 11, the grid cell permeability for the vasculature is *K*_vas_ = 0.02^2^/8 cm^2^ = 5.0× 10^-5^cm^2^ = 5.0 × 10^6^mD.

*Tissue permeability* is calculated according to the number of sinusoids per grid cell. The radius of a single sinusoid is approximately 3μm [4], and so the permeability of a single sinusoid is approximately *K*_sin_ = 3^2^/8 = 1.125μm^2^. The volume of a single human lobule is approximated as 0.001 cm^3^ [2], where there are approximately 43 000 sinusoids per lobule, giving a total lobule permeability of *K*_lob_ = 1.125μm^2^ × 43 000 = 4.8 × 10^3^ μm^2^ = 4.8 × 10^6^ mD. In our example, each grid cell has a volume of 0.04 × 0.04 × 0.04cm = 0.000064cm^3^, which is 0.064 times the size of a human lobule (i.e., a dog lobule is much smaller than a human lobule). Thus, in our model, the permeability of each tissue cell is approximated as *K*_tiss_ = 0.064 × 4.8 × 10^6^mD = 3.0 × 10^5^mD. To obtain a more appropriate permeability value for the tissue space, we perform a series of flow simulations with a predefined (and realistic) pressure drop. For each simulation, we decrease the permeability until we obtain a desired flow rate for our liver structure (permeability and pressure values used in the simulation of blood flow are summarized in Table 2). We calculate the porous volume of our liver structure (shown in Fig 3) to be 84.7cm^3^, and so we suggest the flow rate of approximately 85cm^3^/min, as this rate would allow the liver to refresh itself in approximately 1 minute.

#### Multicomponent flow and metabolism

For multi-component flow, our model tracks the compositions (molar or mass fractions) of all components in the fluid. Here, we focus on the injected drug paclitaxel (PAC) and the phase 1 transformed metabolite 6-hydroxypaclitaxel (PAC-OH). Paclitaxel is an anti-cancer chemotherapy drug used in the treatment of a variety of cancers, including breast, ovarian, lung, bladder, prostate, melanoma, esophageal, as well as other types of solid tumor cancers [37].

For drugs and metabolites, in addition to convective transport (as described in Eq 8), we can also consider a diffusive flux contribution given by

$$J_{ik}^{d}=D_{ik}\nabla_{k}(\rho x_{i}).$$ (12)

Here, $J_{ik}^{d}$ is the molar diffusive flux of component *i* in the direction of *k*, where *d* stands for diffusive flux. The parameter *D*_*ik*_ is the diffusion constant of the species *i* in the *k*-direction. The diffusion constants here are based on those values determined by [2]. In the study of Rezania, diffusion constants were determined for sinusoids and tissue within a single liver lobule. Here, the authors found the effective diffusion of PAC in sinusoids to be 4.2×10^-10^m^2^/sec, and for tissue to be 4.2×10^-11^m^2^/sec. An identical value for PAC-OH is used, since PAC-OH is similar in size to PAC. Here, we assume that diffusion through vasculature is similar to diffusion through sinusoids, and so we define diffusion *D*_vas_ = 4.2×10^-10^m^2^/sec, for both PAC and PAC-OH. To calculate the diffusion rate within tissue cells, we use a similar approach used to calculate the porosity of the tissue cells. In particular, we assume that each tissue grid cell is approximately 8/9 tissue and 1/9 sinusoids. Using the diffusion rates described by [2], we find that the effective diffusion for tissue is *D*_tiss_ = (1/9)×4.2×10^−10^ m^2^/sec + (8/9)×4.2×10^−11^ m^2^/sec = 8.4×10^−11^ m^2^/sec.

In previous work, the drug PAC was used as a reactive tracer, and its phase I metabolism was modeled using the general formula for one PAC molecule being transformed into the metabolite PAC-OH by the cytochrome P450 isozyme CYP2C8 (CYP) [40].

PAC+CYP⟶PAC-OH+CYP (13)

In general, there are different CYP450 enzymes, where the expression of such enzymes vary between different individuals [41]. Furthermore, lobules are characterized by metabolic heterogeneity, being composed of three major zones: zone I (the periportal zone), zone II (the transition zone), and zone III (the pericentral zone), where CYP450 enzymes are located in the pericentral region only [17]. Such analysis is appropriate at the lobule scale (see our earlier work [4]). At the current full liver scale, we assume that such metabolism takes place uniformly in each the tissue grid cells.

Here we base our reaction parameters on the work of Vaclavikova et al. [37], where they measured directly PAC conversion to PAC-OH kinetics based on the activity of the CYP2C8 enzyme. Thus, drug uptake and elimination is considered to be a single-step saturable process that follows Michaelis-Menten-type kinetics [42] such that

$$\frac{d(\rho x_{i}(t))}{dt}=\frac{v_{max}\rho x_{i}(t)}{K_{m}+\rho x_{i}(t)},$$ (14)

where *v*_max_ is the maximum rate of reaction (in units of molar fraction/min) and *K*_*m*_ is the half saturation constant. Similar to the effective diffusion rates, we determine values for *v*_max_ and *K*_*m*_ based on those determined in [2]. In that particular study, *v*_max_ = 0.06 μM/min within the liver lobule hepatocyte cells. For our model, we define *v*_max_ = (1/9)×0 + (8/9)×0.06μM/min = 0.0533μM/min. Similar to [2], we set the initial injection concentration of PAC to be *C*_*0*_ = 1 μM (blood with a relative composition of 1 micro-gram of PAC), and set the half saturation constant to *K*_*m*_ = 10 μM. All parameters used in the simulation of flow and reaction are summarized in Table 2.

To give some perspective on the numerical details of our approach, in S2 Appendix, we compare some of the numerical parameter choices used in our dog liver model with parameters from the mouse liver model of [9]. The approach of [9] is different from ours as they choose a much larger vasculature bifurcation level, but the number of active grid nodes is two orders of magnitude smaller than ours (see S2 Appendix).

### Description of vascular structure by Strahler order

Here, we briefly outline a method used to describe the fractal geometry of vasculature. In particular, it has been determined that vasculature structures, such as the ones described here, are likely to contain fractal properties. Previous studies have calculated parameters, based on the fractal nature of the liver vasculature system, for vasculature structures created using computational approaches (similar to ours), as well as from vasculature structures derived from real data (from either CT scans or corrosion casts) [31]. Such information is important, as it can be used to compare fractal properties of computationally generated vasculature with real vasculature. Interestingly, in the study of [31], the authors also calculate similar parameters based on the fractal nature of vasculatures created in 2D. Thus, we can make comparisons between the fractal properties found in [31] with similar properties described by our 2D vasculature.

The vasculature in [31] were generated using the method of Global Constructive Optimization (GCO), a method similar to that of CCO. GCO is more computationally efficient than CCO, where only local changes are made to the existing tree. Also, optimality conditions and radii relationships can be explicitly introduced. However, these explicit introductions lead to unrealistic looking vascular structures that persist until the end of the vasculature tree generating process.

**Fig 4:**
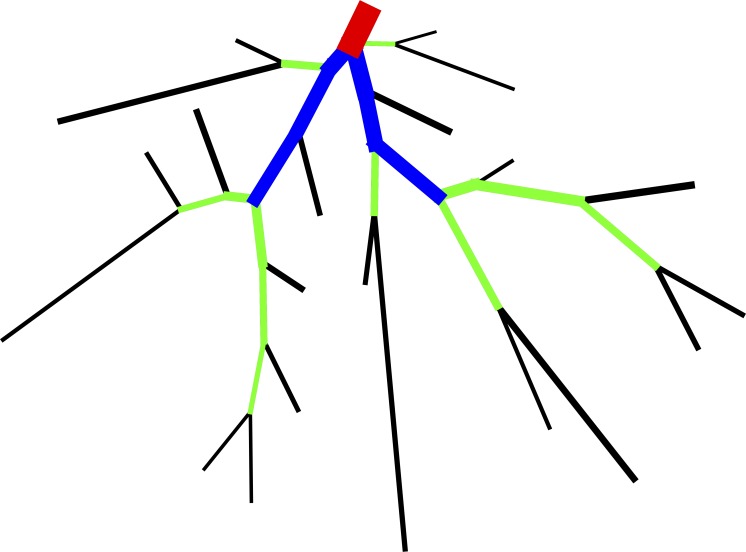
An example of Strahler ordering. Here, black segments are of order 1, green order 2, blue order 3, and red order 1.

The fractal based parameters defined in [31] are defined according to a Strahler ordering scheme, which only requires information regarding the topology of the vasculature tree. It particular, Strahler order is a way of assigning orders to all branches in a vasculature system, where terminal branches are given the lowest order and the root branch is given the highest. Ordering is defined in the following way: First, an order of one is assigned to all terminal segments. Then, working iteratively up the vasculature tree, the order is incremented if two daughter segments having the same Strahler order connect. Fig 4 shows an illustration of the Strahler ordering process.

The three parameters of interest are the Strahler branching ratio (*br*), the Strahler radius ratio (*rr*), and the Strahler length ratio (*lr*). The Strahler branching ratio is the ratio of branch numbers between subsequent Strahler orders. Similarly, the *rr* is the ratio of the mean segment radius between the different orders, while *lr* is the ratio of the mean segment length between the different orders. Results of these parameters calculated for our 2D vasculature are summarized in Table 3. Also, results for the parameters calculated in [31] are summarized here.

**Table 3:** Parameters based on the Strahler ordering of vasculature in 2D. Parameters calculated based on the method of CCO (results from this paper) and GCO [31].

	Branching ratio	Length ratio	Radius ratio
2D portal (CCO) (N = 5)	3.80	1.4	0.66
2D hepatic (CCO) (N = 5)	3.9	1.28	0.68
Planar (GCO) (N = 8)	3.97	1.86	1.55

## Results

In this section, we show results for non-reactive and reactive flow simulations on a simple domain. In particular, we consider the domain described by the 3D structure illustrated in Fig 3A. We would like to stress here that, because we are not considering a realistic 3D liver domain, our results only illustrate how this method is applied. In this first section, we examine the flow rate and the distribution of the drug PAC through the generated 3D structure when we neglect metabolism (i.e., there is no reaction in the tissue grid cells). In the second section, we examine the distributions of PAC, as well as its metabolite PAC-OH, in all grid cells.

### Non-reactive flow on dog liver

To generate a flow (as described in Eq 8), we define a pressure drop across the generated vasculature. Here, we define an input pressure of 104.40kPa at the portal vein root (at the location ‘Prodn_2’ shown in Fig 3) and an output pressure of 101.8kPa at the hepatic vein root (at the location ‘Injtr_1’ as shown in Fig 3). Subtracting atmospheric pressure from these values, we obtain a pressure of 3.075kPa (23.06mmHg) at the portal vein root and a value of 0.475kPa (3.56mmHg) at the hepatic vein root. The total pressure drop is approximately 19.5 mmHg. In real dog livers, the total pressure drop is smaller, generally ranging between 8 and 10 mmHg [12] [13], but can be slightly higher for some dogs [13].

**Fig 5:**
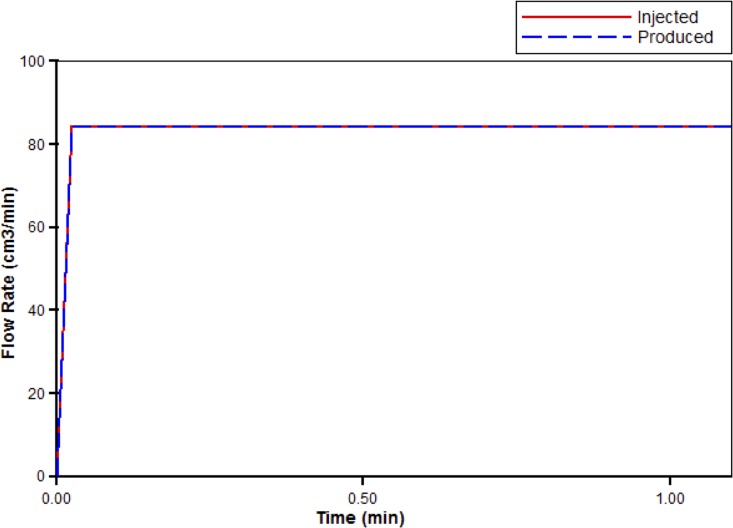
Injected flow (PAC) steady-state flow across the liver structure with short-time flow transient.

Next, blood with a relative composition of one micro-gram PAC per gram of blood (1.8×10–8 mole fraction) is injected at the site of the portal vein root. Fig 5 illustrates a steady-state flow rate of 84.3cm^3^/min. From this figure, we see that it takes a very short time for the flow to reach this steady state.

**Fig 6:**
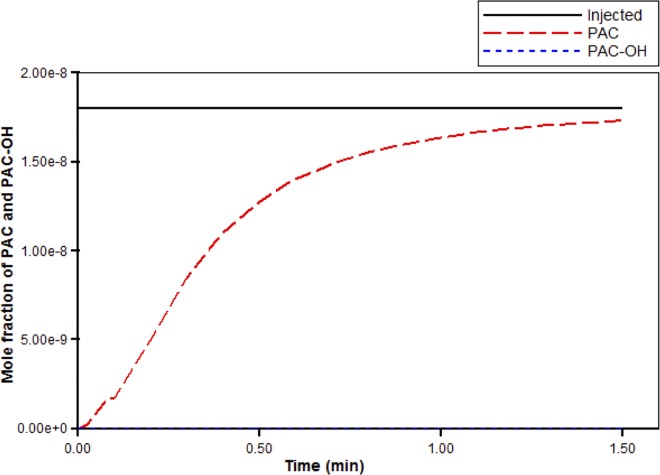
Non-reactive PAC drug propagation across the hepatic root.

Fig 6 illustrates the injected drug value, along with the propagation of drug PAC across the liver. In particular, it illustrates the amount of PAC exiting at the hepatic vein root over time. Here, we see that it takes just over 1.5 minutes for PAC to reach its injected value at the site of the hepatic vein root. This is expected since the value of the flow rate is slightly smaller than that of the porous volume, and so it should take over a minute for PAC to completely reach its injected value. For completeness we also show the PAC-OH propagation, which of course is zero for all time since we are not considering reactive flow in this case.

**Fig 7:**
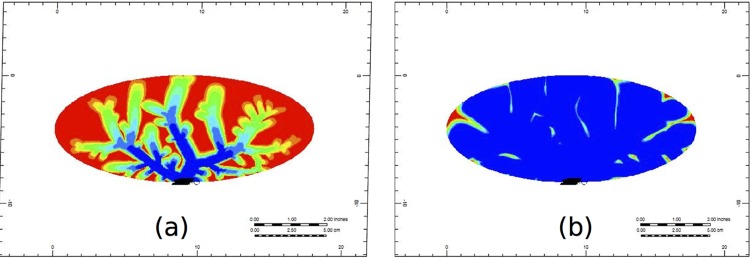
Non-reactive PAC profiles through the cross-section of the portal vein vasculature at (a) 0.1 minutes and (b) 1.5 minutes.

**Fig 8:**
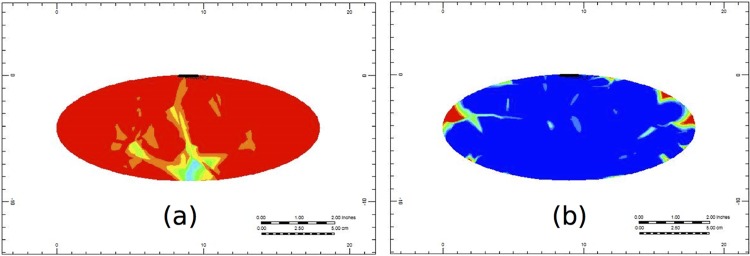
Non-reactive PAC profiles through the cross-section of the hepatic vein vasculature at (a) 0.1 minutes and (b) 1.5 minutes.

**Fig 9:**
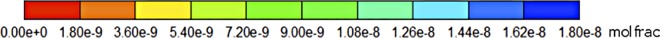
Color bar representing concentration of drug (or metabolite).

To examine the evolution of PAC across the 3D liver structure, we first show the evolution of PAC through cross sections of the portal vein and the hepatic vein vasculatures in Figs 7 and 8, respectively. Fig 9 represents the color bar used for all following figures.

In Fig 7A, we see that after a short time (at 0.1 minutes), the PAC has made its way almost completely through the portal vein vasculature structure, and at this time it has just reached the hepatic vein structure (illustrated by Fig 8A). By 1.5 minutes, the portal vein and the hepatic vein cross-sections are almost completely covered by PAC (illustrated in Figs 7B and 8B, respectively). Here, PAC quickly fills the vasculature structure, and only at later times do we see the PAC spread through the rest of the liver’s tissue.

We also tested the contribution of diffusion to the distribution of PAC throughout the liver, by a comparative simulation with the PAC diffusion constant set to zero. However, visually significant changes in PAC distribution were not observed (results not shown).

**Fig 10:**
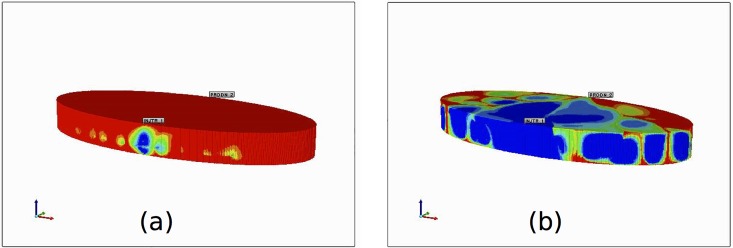
Non-reactive 3D PAC profile over the entire liver structure at (a) 0.1 minutes and (b) 1.5 minutes.

The full 3D PAC distribution is shown in Fig 10. Here, we see that after 1.5 minutes there are still many places on the surface of the liver where the PAC has not yet reached, highlighting that the perfusion of PAC is not homogeneous. One reason for this it that we only generate a small number of vessel segments for each vasculature (as compared to a real liver). If more segments are generated, it is more likely that the PAC will be distributed to more places at the liver surface.

### Reactive flow on dog liver

**Fig 11:**
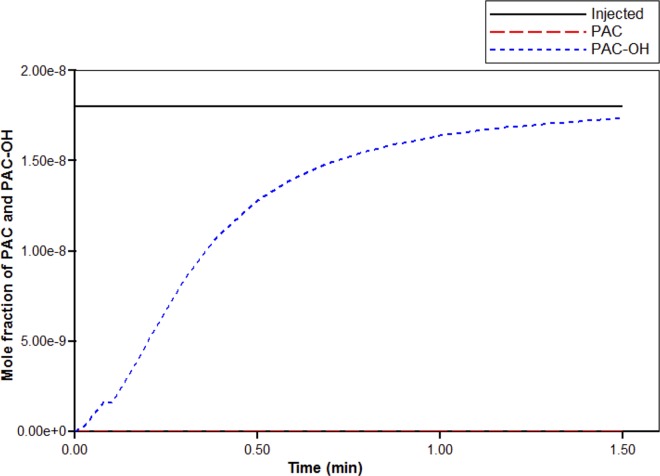
PAC-OH and PAC drug propagation across the hepatic vein root.

Here we show results for reactive drug flow. In particular, we consider the effects of PAC drug metabolism. Fig 11 illustrates PAC and PAC-OH propagation across the liver structure, as well as the injected value of PAC. Using the reaction parameters given in Table 2, we see that PAC is completely metabolized in the tissue cells, so that the only contribution of the drug exiting the hepatic vein is PAC-OH. Similar to PAC elimination for the non-reactive case (see Fig 6), it takes about 1.5 minutes for the drug to reach its injected value at the hepatic vein root.

**Fig 12:**
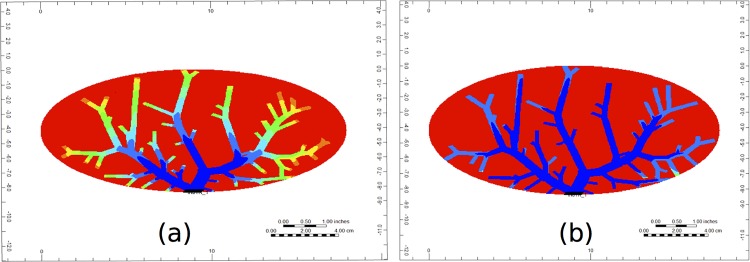
Reactive PAC profiles through the cross section of the portal vein vasculature at (a) 0.1 minutes and (b) 1.5 minutes.

**Fig 13:**
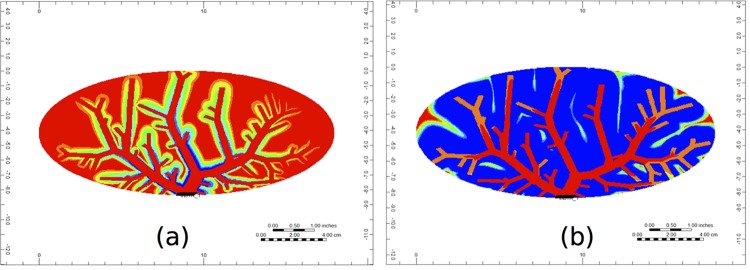
Metabolite PAC-OH profile through the cross section of the portal vein vasculature at (a) 0.1 minutes and (b) 1.5 minutes.

Figs 12 and 13 illustrate PAC and PAC-OH distribution through the cross-section of the portal vein vasculature structure, respectively. Unlike the non-reactive case (shown in Fig A), Fig 11A illustrates that there is no spread of PAC from the vasculature. This is because the metabolism for PAC is very fast. In particular, as soon as PAC enters a tissue grid cell, it is quickly metabolized. Fig 13A shows how PAC-OH is quickly produced, outlining the portal vein vasculature after 0.1 minutes. After 1.5 minutes, the PAC-OH has almost completely covered the portal vein vasculature, except in the portal vein itself (see Fig 13B).

**Fig 14:**
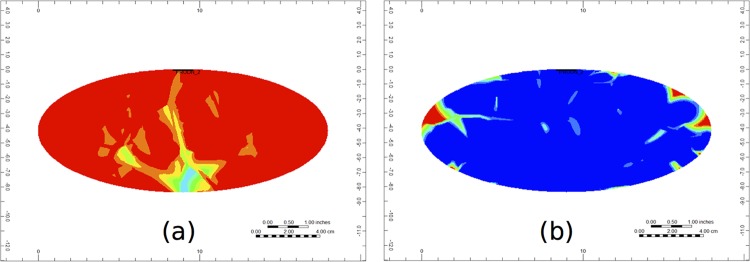
Metabolite PAC-OH profile through the cross-section of the hepatic vein vasculature at (a) 0.1 minutes and (b) 1.5 minutes

Fig 14 shows the PAC-OH distribution through the cross-section of the hepatic vein vasculature. At 1.5 minutes, the PAC-OH has almost completely covered this cross-section (see Fig 13B).

**Fig 15:**
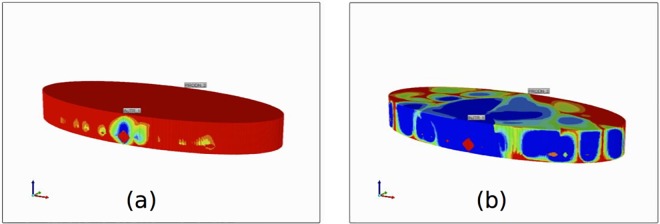
Reactive 3D PAC-OH profile over the entire liver structure at (a) 0.1 minutes and (b) 1.5 minutes.

Fig 15 shows the 3D PAC-OH distribution. Similar to Fig 10 for PAC, the reacted PAC-OH has not completely covered the entire liver structure, although almost all PAC has reacted to PAC-OH. Like the previous case, this can be explained by the relatively small number of vessel segments that are generated for the liver structure.

### Comparison of fractal parameters with other studies

Here, we compare our ‘in silico’ 2D vasculature created by CCO with ‘in silico’ 2D vasculature created with GCO [31]. We illustrate that our 2D results for branching ratio and length ratio are consistent with the 2D results described in [31].

As shown in Table 3, the parameters calculated for our 2D portal and 2D hepatic liver have similar branching ratios, as well as similar length ratios, as those parameters calculated for 2D vasculature created using the method of GCO [31]. Our branching ratio is quite a bit smaller, and is probably due to the fact that the relationship between radii are explicit inputs for the model in [31], and are not explicit inputs in our modeling approach.

## Discussion and Conclusions

In this paper, we have outlined a method for generating a vasculature structure in 2D using the method of CCO, and extending it to a pseudo 3D domain where the branching angles of each vasculature are in 2D. We have also described how one can simulate blood flow and drug metabolism on each generated structure. The purpose of this paper is to outline the methodology for this modeling approach, and in future work we will extend our model to a fully 3D representation of a liver. A key benefit of our spatio-temporal modeling approach is that we can describe the dynamics of blood and drugs on complex vascular structures, and describe blood and drug dynamics at small time scales. In a clinical setting this can be very useful, as one can track the evolution of a drug through the liver from the instant it is administered.

A limitation of our present model is that it is essentially a 2D model (3D with branching angles in 2D). This avoids the issue of generating two non-overlapping vasculatures simulataneously in 3D (currently under development). However, this does allow a clear description of the methodology.

With the exception of the input pressure drop, the parameters used in the creation of the vasculature structures (the 2D images shown in Fig 1) are similar to those for dogs. Here, our pressure drop (the pressure difference between the root of a tree and its terminals) is very low, another limitation of our modeling approach. As explained in Methods and Models, one reason for this is that the number of bifurcations used is relatively small (only 50 bifurcations are used in the example in this paper). If we increase the number of bifurcations, keeping all other parameters fixed, the root radius will increase. To offset the increase in root radius, we can increase the total pressure drop (effectively increasing the total resistance of the tree), to a higher value. However, this does not fully explain why we used the low pressure drops, since increasing the bifurcation value only increases the root radius by a very small amount (results not shown), a result that is consistent with that of Schreiner [10].

A second contributing factor to why our pressure drops are low is based on the choice of the bifurcation parameter *γ*. In this paper, we choose *γ* = 3 (as was initially used in the creation of vasculature in the heart [10]. However, if we choose *γ* = 2.55, as was used in [28], we are able to use much more reasonable pressure drops in the creation of each vasculature (results not shown). By decreasing the value of *γ*, the corresponding decrease in the radius of each new generation of segments is larger than in the case when *γ* = 3. To offset this decrease, we increase the pressure drop to obtain the desired root radius at the end of the vasculature generation. This finding suggests that different organs may require different bifurcation parameters. In fact, in a recent study by Schwen et al. [30], experimental data has found that the value for gamma in humans is approximately equal to 1. This suggests the need for further studies to test whether this parameter varies between organ type (and perhaps animal type).

Presently, we generate vasculature structures with only 50 bifurcations, where we implicitly account for smaller vessels in the tissue space (sinusoids) using an appropriate upscaling technique to define an average grid permeability. The addition of many more vessels will require that we define vessels with diameter smaller than the current grid spacing. To remedy this issue, we are working to introduce a dual continuum approach, implemented by STARS [14]. Using this approach, porosity and permeability values representing both sinusoid and tissue continua in a single grid cell can be defined, and so this method can be used to implicitly describe both the vessels that are smaller than the dimension of the grid cell, as well as surrounding tissue that fills the same grid cell. Alternatively, we could define smaller grid cells, however this would lead to greater computational expense.

Simulations on our generated liver structure produced a steady-state flow rate of 84.3cm^3^/min through both the portal vein root and the hepatic vein root. In an average sized dog liver, blood flows at an approximate rate of 240cm^3^/min through the portal vein root, and at a rate of 390cm^3^/min through the hepatic vein root [5]. We expect that, by generating fully 3D vasculature, the flow rates will be more realistic because we will describe larger vasculature structures (by generating more vessel segments), and we will consider a realistic 3D liver domain. Models of 3D vascular trees have previously been developed [23] [30], but it is difficult to fully describe hepatic clearance on such structures, as the construction of two non-overlapping vasculature is not easy to generate [25].

A second result of our simulations was that the addition of diffusion to the model resulted in an insignificant change in the overall distribution of PAC over the liver structure, using our current estimate for the PAC diffusion constant. This result might suggest that blood flow through the liver at the organ scale is primarily a convective process, where diffusion has a minimal effect. Interestingly, in similar studies of blood flow at the lobule scale, results suggest that the inclusion of diffusion significantly changes the distribution of PAC and PAC-OH through the liver structure [2]. Currently, we have neglected the complex architecture of the individual tissue cells (comprised of lobules), where blood flows in an anisotropic manner through each of the lobules sinusoids. A future goal of this project would be to obtain a complete (and more realistic) description of blood flow and drug metabolism at both scales of the liver (organ and lobule). In particular, we would like to design a multi-scale approach, combining the lobule scale model described by Rezania et al. [2] [3] [4] with our modeling approach. In the model of Rezania et al., lobules are treated as hexagonal prisms, where flow and metabolism are defined according to the proper zonation of a real lobule.

It is also interesting to note the heterogeneity of the PAC-OH distribution on the surface of the liver (shown in Fig 13). A real liver is comprised of four main lobes, where each lobe is perfused according to how the vasculature extends into each of these lobes. In general, blood is not perfused uniformly over the liver surface, and this model highlights an example of this.

A final point we make is that the pressure drops and flow rates used in the generation of the vasculatures are different from the pressure drop and generated flow rate obtained from the STARS simulations. In the former, we are only concerned with describing flow through the generated vasculature itself, and not with describing flow throughout the entire organ’s domain. When we simulate blood flow in the STARS model, we take into account the porosity and permeability of both the vasculature and the tissue space, effectively and correctly adding additional resistances to the flow problem.

The results obtained in this study are representative of blood flow and drug metabolism on a simple 3D domain. It is important to note that the structure generated here is only an approximation of a liver, and that the aim of this paper is only to illustrate a method that can be used to describe hepatic clearance on any 3D structure. In this paper, we generate 3D vasculature with branching angles in 2D. As such, our method can produce artifacts in flow patterning and metabolism that would not be seen in a fully 3D case. In particular, our model allows for directed flow between the portal and hepatic vascular structures. To reduce this effect, we reduce the transmissibility of flow in the vertical direction, so that the majority of the blood flows almost completely through the portal vasculature structure before entering the hepatic vasculature system. Also, Figs 12 and 13 illustrate a very small reaction area around the generated vasculature. By generating more vessel segments, as well as using the dual continuum approach mentioned above, we will be able to generate a structure where much more of the tissue space is actively involved in metabolism. A simple example of dual continuum modeling of subgrid level vasculature is presented as S3 Appendix. Also, the reaction rate used (see Table 2) is an average value for a tissue grid cell of 0.04 cm size. Such a reaction constant is an effective value and a more consistent up-scaling procedure must be employed to better quantify its exact value. Furthermore, non-homogeneous distribution of metabolism at the full liver scale, (eg due to stenosis, see Schwen et al [43][7]) is well known and this could be easily incorporated into our model via non-homogeneous lobule properties.

Furthermore, we have presented and discussed several issues required to convert a lattice-free vasculature generation method to a lattice-based discretized flow model describing reactive drug transport. In future studies, we will generate more realistic 3D models, as well as models for human liver. In fact, similar computational methods are being used to create 3D vasculature structures [28][8][24][9]. Currently, we are working on generating two non-overlapping vasculature in 3D (an example is illustrated in S1 Appendix). In reality, both portal and hepatic vasculature grow very closely around each other, and it is difficult to simulate this interwoven construction, so as not to have the vasculature merge together. Similar issues have been described by other authors who use computational approaches to generate liver vasculature [30][10]. Also, the anatomical data required to accurately describe the complete architecture of the human liver vasculature is lacking, and future collaborations with experimentalists who can obtain such data would be useful to increase the realism of our model. In particular, we would like to compare our generated vasculature with data from CT scans or corrosion casts, as was done by [28]. As a first step to model validation, we are able to compare fractal properties of our 2D structures constructed using CCO with the fractal properties of 2D vasculature structures created using GCO [31][11]. The fractal properties of real vasculature (obtained from corrosion casts) are also summarized in [31][11], and it will be useful to compare our future 3D results with these results.

As a final note, to fully describe hepatic clearance, we will not only require a liver model, but also an organism-level pharmacokinetic model. This will require connecting our spatially-defined liver model to an organism-level pharmacokinetic model, perhaps similar in approach to Johnson *et al*. [44][12] who utilized a three compartment body-scale recirculation model.

## Supporting Information

S1 Appendix An example of 3D vasculature created using CCO. (DOCX)

S2 Appendix Parameter comparison. (DOCX)

S3 Appendix Dual Continuum Model of Subgrid Level Vasculature. (DOCX)
