# Supplementary material for: Building a 3D Virtual Liver: Methods for Simulating Blood Flow and Hepatic Clearance on 3D Structures
Source: PLoS One. 2016 Sep 20;11(9):e0162215. doi: 10.1371/journal.pone.0162215 (PMC5029923; doi:10.1371/journal.pone.0162215)
Supplement: S1 Appendix — (DOCX) [file pone.0162215.s001.docx]

**S1 Appendix: An example of 3D vasculature created using CCO.**

Example of 3D vasculature generated by method of CCO on liver-like domain.


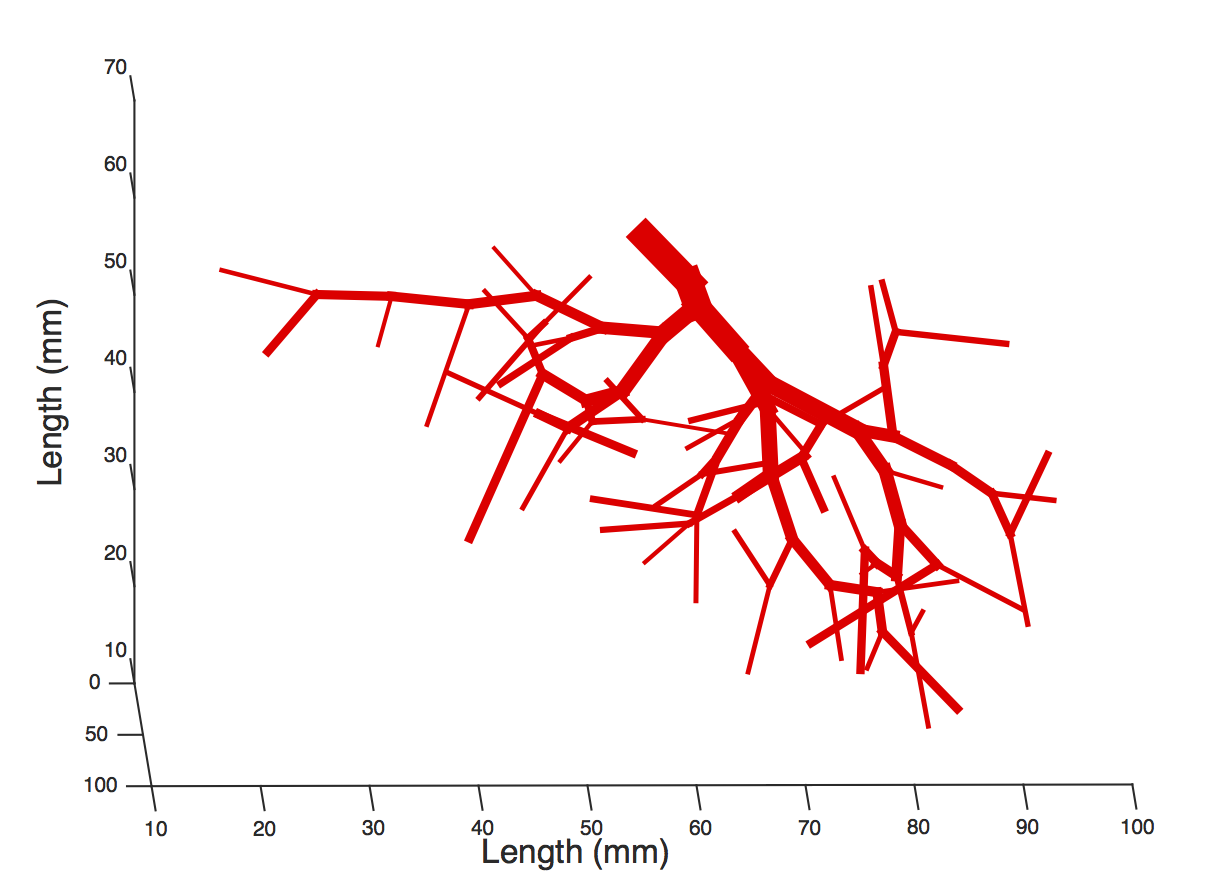


**Fig. S1.** Example of 3D vasculature created on liver-like domain. Here, *Q*_tot_ = 330 ml/min, Δ*P* = 2.50 mmHg , and *r_0_* = 0.25cm. The number of bifurcations (BifNum) is 50, and the bifurcation parameters used in *γ* = 2.55.


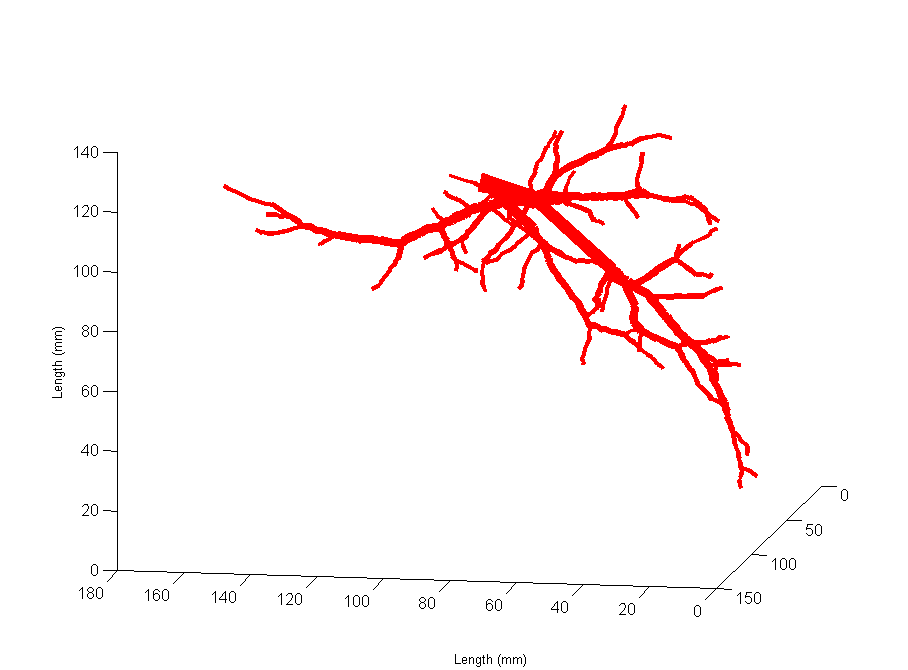


**Fig. S2**. Example of 3D vasculature extracted from Schwen et al. [1] showing major portal vein vasculature for visual comparison. Only radii larger than ~ 0.75 mm are plotted.

# References

x

| 1. | L. Schwen, A. Schenk, C. Kreutz, J. Timmer, M. Bartolomé Rodríguez, L. Kuepfer et al., "Representative Sinusoids for Hepatic Four-Scale Pharmacokinetics Simulations," PLoS ONE, vol. 10, no. 7, p. e0133653, 2015. |
| --- | --- |

x
