# Supplementary material for: Building a 3D Virtual Liver: Methods for Simulating Blood Flow and Hepatic Clearance on 3D Structures
Source: PLoS One. 2016 Sep 20;11(9):e0162215. doi: 10.1371/journal.pone.0162215 (PMC5029923; doi:10.1371/journal.pone.0162215)
Supplement: S2 Appendix — (DOCX) [file pone.0162215.s002.docx]

**S2 Appendix: Parameter comparison**

Here we compare the numerical parameter choices used for a discretized model of mouse liver [1] with our model (approximation to dog liver).

| **Parameter** | **Mouse Liver** | **Dog Liver** |
| --- | --- | --- |
| *Liver size* | 1 cm^3^ | 256 cm^3^ |
| *Perfusion rate* | 2.1 cm^3^/min | 84.3 cm^3^/min |
| *#leaves (= bifurcation No.)* | 800 | 50 |
| *Active grid nodes* | 49,114 | 5,103,000 |
| *HHS (grid size)* | 0.028cm × 0.028cm × 0.028cm | 0.04cm × 0.04cm × 0.04cm |
| *Average timestep size (variable)* | 8.33×10^-4^ min (0.05 sec) | 0.05 min |

Table S2: Comparison of parameter choices for our model and the liver model of Schwen et al. [1]

# References

x

| 1. | Schwen L, Krauss M, Niederalt C, Gremse F, Kiessling F, Schenk A, et al. Spatio-temporal simulation of first pass drug perfusion in the liver. PLOS Computational Biology. 2014; 10(3): p. 1-18. |
| --- | --- |

x
