# Supplementary material for: Building a 3D Virtual Liver: Methods for Simulating Blood Flow and Hepatic Clearance on 3D Structures
Source: PLoS One. 2016 Sep 20;11(9):e0162215. doi: 10.1371/journal.pone.0162215 (PMC5029923; doi:10.1371/journal.pone.0162215)
Supplement: S3 Appendix — (DOCX) [file pone.0162215.s003.docx]

**S3 Appendix: Dual Continuum Model of Subgrid Level Vasculature**

This appendix introduces dual continuum modelling of subgrid level vasculature as a computationally efficient attractive alternative to fine grid representations of higher generation levels of vasculature in on-lattice flow models. The essential problem is how to convert reference fine grid **intrinsic properties** to **effective properties** for the dual continuum model for choices of grid size larger than the reference grid (upscaling).

Here we will consider a region of space of 2 cm × 2 cm × 6 cm and place a vasculature tube of radius 0.5 cm and length 6 cm in the middle of this region. Two flow models of this system will be constructed and compared.

First a fine grid Cartesian model of this flow system is constructed utilizing an 8 × 8 × 3 grid system (with grid size 0.25 cm × 0.25 cm × 2 cm). This base case is termed a “single porosity (SP)” model with one set of parameters per grid cell and which utilizes **intrinsic parameters** of tissue and vasculature in the respective regions of space inside and outside of the vasculature and specifically discretizes each region. Here we approximate the vasculature “tube” as a high flow region of a Cartesian cross section of grid blocks. This representation slightly underestimates the cross section area of an equivalent radial tube.

To construct such models we will follow the assumptions for **intrinsic property** parameters as chosen for our earlier flow models of the liver organ. These have been justified in earlier discussions and are summarized in Table 1.

| **Parameter** | **Value (SI units)** | **Value (STARS unit)** | **Intrinsic properties** |
| --- | --- | --- | --- |
| *𝜙_vasc_* | 0.9 | 0.9 | Vasculature porosity |
| *𝜙_tiss_* | 0.2980 | 0.2980 | Tissue porosity |
| *K_vasc_* | 2.5×10^-9^ m^2^ | 2.5×10^6^ mD | Vasculature permeability |
| *K_tiss_* | 1.0×10^-9^ m^2^ | 1.0×10^6^ mD | Tissue permeability |
| *SW_vasc_* | 1.0 | 1.0 | Saturation ratio in vasculature |
| *SW_tiss_* | 0.33 | 0.33 | Saturation ratio in tissue |

Table 1: **Intrinsic** tissue and vasculature properties used in fine grid SP case.

The second “dual continuum” or “dual permeability (DK)” model is a coarse grid 1 × 1 × 3 system (with grid size 2 cm × 2 cm × 2 cm) which specifies two values at every grid location (for tissue and vasculature) and utilizes **effective parameters.** These effective parameters can be calculated by recognizing the relative areas and volumes associated with the tissue and vasculature in each grid cell. For a tube of radius 0.5 cm in a grid cell of 2 cm × 2 cm cross section, the relative areas and volumes for flow for vasculature and tissue are

$$\left(\frac{A_{tube}}{A_{grid}}\right)_{vasc}= \frac{0.5\text{cm }\times\text{ }0.5\text{cm }\times\text{ π}}{2\text{cm }\times\text{ }2\text{cm}}\text{ }=\text{ }0.19635$$

$$\left(\frac{A_{tube}}{A_{grid}}\right)_{tiss}= 1-\left(\frac{A_{tube}}{A_{grid}}\right)_{vasc}=\text{ }0.80365$$

$$\left(\frac{V_{tube}}{V_{grid}}\right)_{vasc}= \frac{0.5\text{cm }\times\text{ }0.5\text{cm }\times\text{ π }\times2\text{cm}}{2\text{cm }\times\text{ }2\text{cm}\times\text{ }2\text{cm}}\text{ }=\text{ }0.19635$$

$$\left(\frac{V_{tube}}{V_{grid}}\right)_{tiss}= 1-\left(\frac{V_{tube}}{V_{grid}}\right)_{vasc}=\text{ }0.80365$$

This then implies that we can calculate **effective properties** for this system as

$$q_{i}^{eff}=q_{i}^{\mathrm{int}}\times\text{relative factor}$$

where shown in Table 2:

| **Parameter** | **Value (SI units)** | **Value (STARS unit)** | **Effective properties** |
| --- | --- | --- | --- |
| *𝜙_vasc_* | 0.1767 | 0.1767 | Vasculature porosity |
| *𝜙_tiss_* | 0.2395 | 0.2395 | Tissue porosity |
| *K_vasc_* | 0.4909×10^-9^ m^2^ | 0.4909×10^6^ mD | Vasculature permeability |
| *K_tiss_* | 0.8037×10^-9^ m^2^ | 0.8037×10^6^ mD | Tissue permeability |
| *SW_vasc_* | 0.1964 | 0.1964 | Saturation ratio in vasculature |
| *SW_tiss_* | 0.2652 | 0.2652 | Saturation ratio in tissue |

Table 2: **Effective** tissue and vasculature properties used in coarse grid DK case.

These calculations ensure that on a (grid block) volume basis, a coarse grid dual continuum model preserves the same average behavior as a fine grid single porosity model (except for the Cartesian versus radial representation of the tube mentioned above).

To make these comments more concrete, some plots comparing the two modelling approaches are presented. Figure 1 shows the single porosity representation of **intrinsic** permeability. The aerial view of the top layer illustrates the higher permeability zone representative of the vasculature in the center of the model, and which penetrates the complete model.


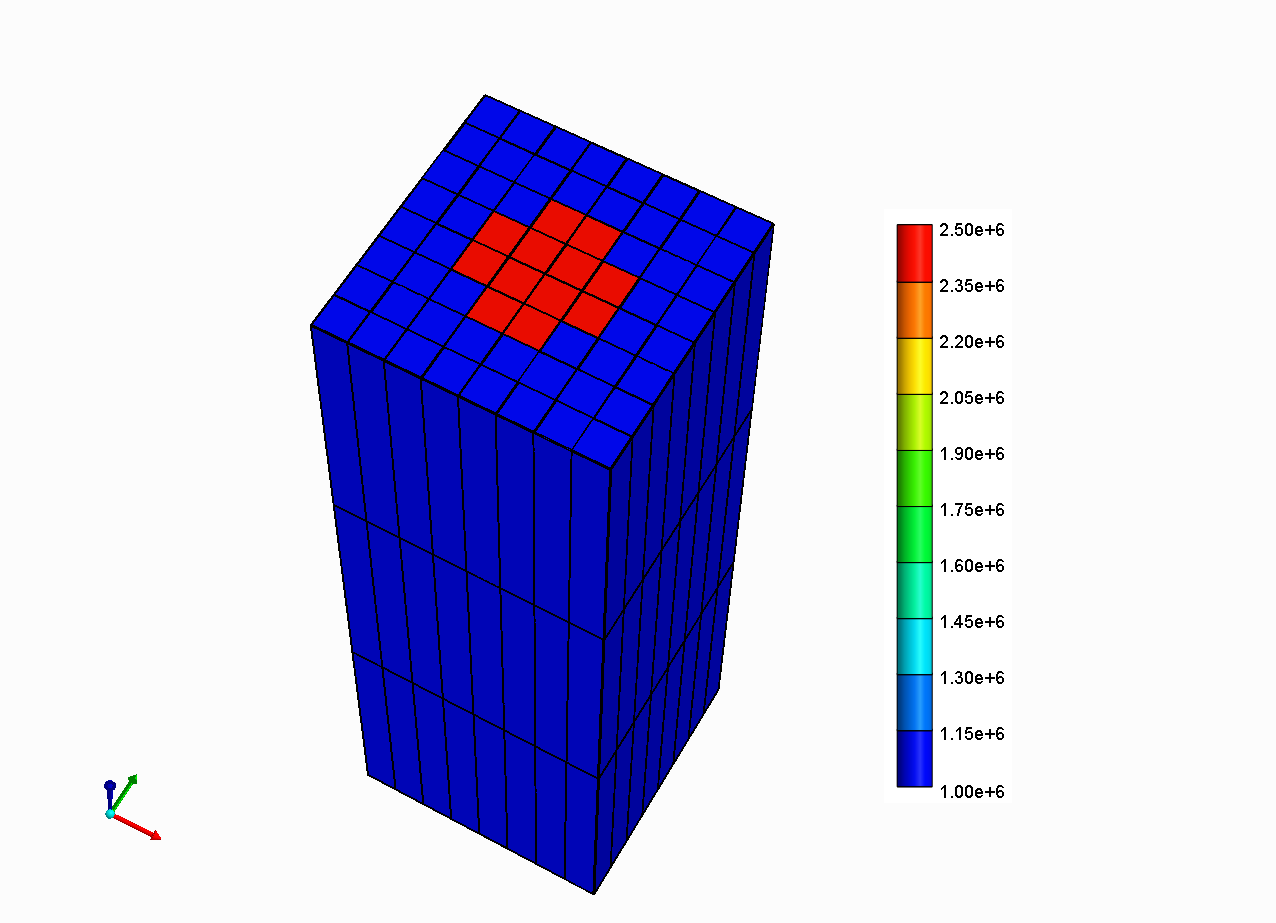


Fig. 1: Single porosity representations of intrinsic permeability distribution, single tube case.

The corresponding representation of the same physical phenomena in a dual continuum approach has two sets of properties, each in coarse grid 1 × 1 × 3 grid. Here the **effective** matrix (i.e. tissue) permeability shows a constant value of 8.04 × 10^5^ mD while the corresponding fracture (i.e. vasculature) effective permeability is also a constant, 4.91× 10^5^ mD.

Although these views of the single porosity versus dual permeability modelling approaches appear different, Table 3 (from the STARS output files) shows the initial volumes in place for the two modelling approaches are consistent. There is a slight difference due to gridding errors discussed earlier – the Cartesian representation of the vasculature for the single porosity model versus the radial volumetrics of the vasculature tube used to determine the dual permeability effective porosities.

|  | **Total** | **Tissue** | **Vasculature** |
| --- | --- | --- | --- |
| **Single porosity (SP) fine grid** |  |  |  |
| Gross formation volume (cm^3^) | 24.000 |  |  |
| Formation pore volume (cm^3^) | 9.8610 |  |  |
| Aqueous phase volume (cm^3^) | 5.9676 |  |  |
| Cell phase volume (cm^3^) | 3.8934 |  |  |
| **Dual continuum (DK) coarse grid** |  |  |  |
| Gross formation volume (cm^3^) | 24.000 | 19.288 | 4.7124 |
| Formation pore volume (cm^3^) | 9.9904 | 5.7477 | 4.2427 |
| Aqueous phase volume (cm^3^) | 6.1394 | 1.8967 | 4.2427 |
| Cell phase volume (cm^3^) | 3.8510 | 3.8510 | 0.0000 |

Table 3: Initial volumes in place: single porosity versus dual permeability cases.

As a check on the flow performance of the two modelling approaches, Figure 2 compares the predicted flow rates in the two models. Since these models are run under the same specified pressure drop (injector pressure 103.0 kPa; producer pressure 101.8 kPa), the resultant flows reflect the effective overall permeabilities for the two models. The observed differences can again be attributed to the Cartesian versus radial representations of the vasculature.


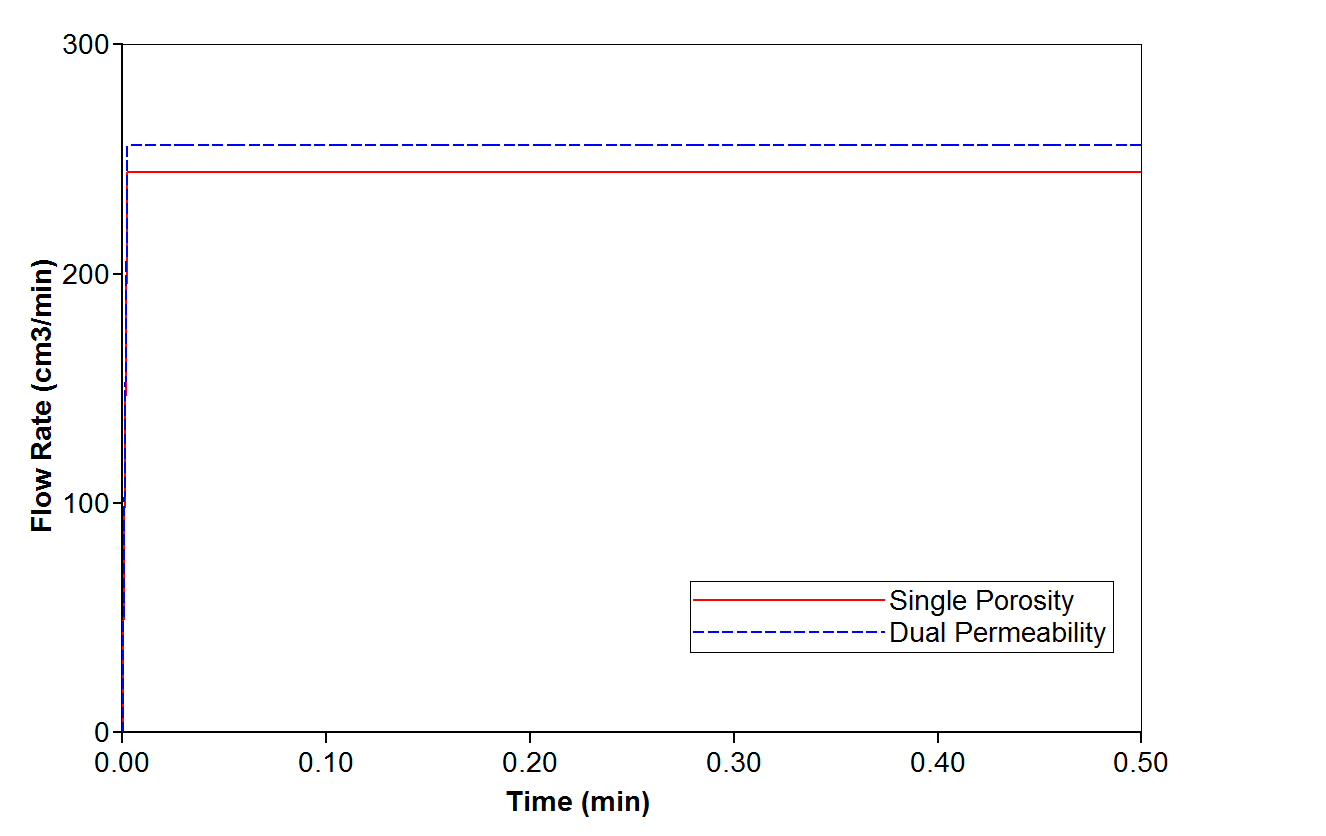


Fig. 2: Flow rate predictions: single porosity versus dual permeability, single tube case.

As a final comparison, evolving PAC profiles in the two models are considered as PAC is injected from the top of the model and later produced from the bottom of the model. Note it is expected that PAC will initially be transported along the higher permeability vasculature, but because of a degree of leakage along the tube, PAC will also appear in the surrounding tissue. Because of continuous PAC injection, ultimately injected PAC concentration should fill all regions of the model. It is the overall time evolution of this process which is of interest.

Produced drug concentration predictions are shown in Figure 3. The two modelling approaches give identical production characteristics.


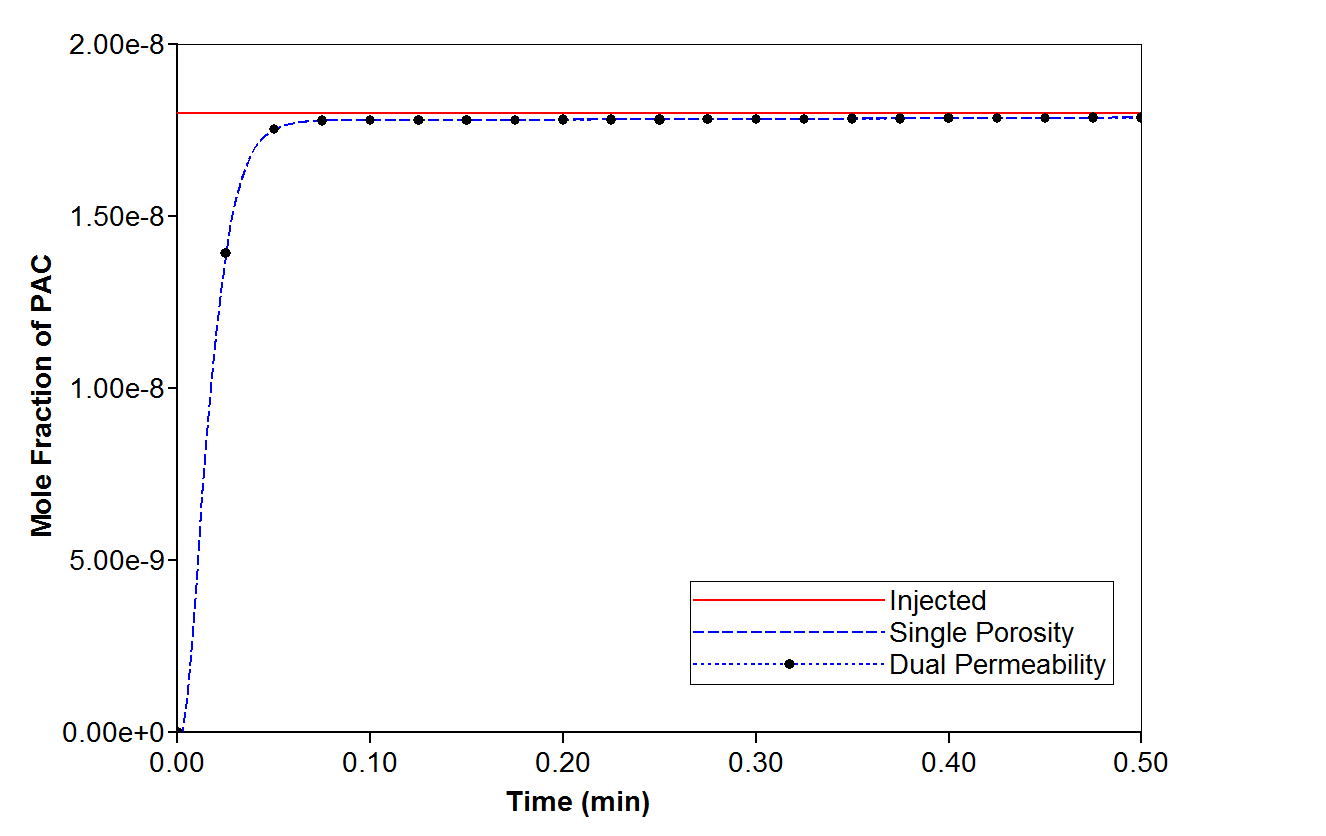


Fig. 3: Produced drug concentration predictions: single porosity versus dual permeability, single tube case.

In summary, aspects of the dual continuum modelling of subgrid vasculature have been described. Such an approach first requires assumed values for intrinsic parameters for porosity, permeability and water saturation for both tissue and vasculature flow types. This, coupled with a knowledge of vasculature radii and the number of tubes for each grid block in the on-lattice flow model (as generated from the off-lattice CCO algorithm), is sufficient to generate a dual continuum model for multiple generations of subgrid vasculature by following the procedure outlined herein.

It is emphasized that the dual continuum model is an attractive computationally efficient approach. For the case studied here, the dual continuum model consists of 2 × (1 × 1 × 3) = 6 grid blocks while the single porosity approach requires 8 × 8 × 3 = 192 grid blocks. An approximate estimate of computational efficiency (e.g. run time speedup) would then be 32 times. This improvement might be expected to be substantially greater when applying this technique to realistic 3D models of the full liver with vasculature.
